# Local knowledge about fodder plants in the semi-arid region of Northeastern Brazil

**DOI:** 10.1186/1746-4269-11-12

**Published:** 2015-02-10

**Authors:** Alissandra Trajano Nunes, Reinaldo Farias Paivade Lucena, Mércia Virgínia Ferreira dos Santos, Ulysses Paulino Albuquerque

**Affiliations:** Departamento de Biologia da Universidade Federal Rural de Pernambuco, Laboratório de Etnobiologia Aplicada e Teórica (LEA), Recife, Pernambuco, CEP: 52171-900 Brazil; Departamento de Fitotecnia e Ciências Ambientais, Setor de Ecologia e Biodiversidade, Laboratório de Etnoecologia, Universidade Federal da Paraíba. Campus II. Centro de Ciências Agrárias, Areia, Paraíba, CEP: 58.397-000 Brazil; Departamento de Zootecnia, Universidade Federal Rural de Pernambuco, Recife, Pernambuco, CEP: 52171-900 Brazil

**Keywords:** Ethnobotany, Ethnobiology, Animal feeding plants, Subsistence livestock

## Abstract

**Background:**

This study evaluated local knowledge of the fodder plants of the Caatinga in northeast Brazil (seasonal dry forest). Specifically, the goal was to catalog local knowledge regarding the use of native and exotic forage plants in two rural communities located in the state of Paraíba (northeast Brazil), to provide information for nutritional investigations and to verify how the knowledge of these resources is distributed.

**Methods:**

The communities were followed for three consecutive years, and interviews were conducted with 44 families (20 men and 24 women). Nine of these individuals were determined by the snowball technique to be key informants who held more specific knowledge about the topic. The data were structured into a database and statistically analyzed.

**Results:**

Overall, 136 plants from 37 families and 113 genera were cited, and the knowledge of men was at a higher level than that of women (p < 0.05). Participants demonstrated a sophisticated knowledge of nutritional characteristics such as nutritional value, palatability, availability and productivity. Native plants were highlighted over the exotic, especially for species of the families Cactaceae, Bromeliaceae and Fabaceae.

**Conclusions:**

The great diversity of plants cited by the informants demonstrates the potential of local vegetation and the importance of traditional knowledge in the research process and in the characterization of forage resources. This diversity also favors the selection of promising species for future biotechnological investigations.

## Introduction

Animal breeding is an ancient practice that represents an important source of subsistence for low-income households worldwide [1]. In Brazil, this activity plays an important role in the local economy, especially in the region of northeastern Brazil, where it accounts for more than 90% of the national flock of small ruminants, such as goats and sheep [2]. In this region, more than 50% of the territory is occupied by typical Caatinga vegetation (seasonal dry forest), which is the largest source of food for these animals [3].

Although it has been recently devastated, the Caatinga offers a good diversity of potential plants for the diet of ruminants [4, 5]. However, the region, which is characterized by a semi-arid climate, has suffered environmental pressures and is affected by a strong influence of climatic seasonality that limits the productive and nutritional potential of the vegetation. The result is that it is difficult to maintain flocks, especially during drought periods [4, 6].

The situation in northeast Brazil mainly affects small farmers and is common in other countries with similar climatic conditions, such as Nigeria and Pakistan [7–9]. Given the growing need to produce human food and to secure subsistence food sources for flocks, there is a growing interest in expanding the knowledge about the fodder plants in these areas [1, 7–11]. The investigation has advanced in the Caatinga [4, 5] to characterize the local natural resources and determine alternatives that may ease the difficulties of people who depend on subsistence farming to survive [12, 13].

In the investigation of food sources in different regions of the world, it is essential to document local knowledge as a valuable resource in the characterization of fodder plants [11, 14, 15]. This information can be captured in local history as well as by the indications of fodder uses in the lists of plants that were generated by ethnobotanical research conducted in the Northeastern countryside [16–18].

With respect to ethnobotanical approaches in northeastern Brazil, there is a gap related to the forage resource evidence in relation to reports published by researchers from other countries [7, 14, 19, 20]. Consideration should be given to prioritizing access to local knowledge to combine scientific knowledge and optimize the process of characterizing the diversity of plants useful for this purpose.

In the last few decades, the union of scientific knowledge with traditional knowledge has been increased through several approaches. This union has revealed the importance of local wisdom in conservation as well as in providing basic information for bioprospecting [21–23].

Ethnobotanical investigations of fodder plants have been developed in African countries such as Ethiopia, Nigeria and Uganda, and elsewhere in Asia, India, and Mexico. These works are guided by approaches that reinforce the importance of local knowledge associated with the use, classification and management of useful plants in animal feed, and in seeking alternatives to reconcile sustainability and conservation of the local biodiversity see [7, 14, 19].

This research aims to analyze the traditional knowledge about the plants used in animal feeding in two rural communities in northeastern Brazil. The work is based on the premise that rural communities in semi-arid environments have an expansive knowledge of the diversity and nutritional potential of plants used as ruminant feed. Our main goals were to identify the species used in animal diets mentioned in both communities, check for differences in the biogeographical origins of the plants and identify the influence of seasonality in the diets of the animals.

Because animal husbandry is an activity performed by men, we evaluated whether there was a difference in knowledge between men and women. Therefore, we evaluated the responses by gender and correlated variables such as age, income and education with the use of fodder plants.

## Materials and methods

### Area of study

The present study has been developed in the semi-arid region of Northeastern Brazil. The region is home to the Caatinga biome, which is considered to be the only Brazilian biome that is exclusive to Brazil. The biome shows distinct edaphoclimatic conditions that create units of differentiated landscape. This situation gives rise to diverse vegetation that is represented by a considerable richness of endemic species [3].

In terms of occupation, the region covers approximately 10% of the national territory and more than 80% of the Northeastern territory, with approximately 970,000 Km^2^ of territorial extension inhabited by more than 20 million people [24].

A considerable part of the population lives in rural areas and historically is linked to agriculture, and particularly to livestock, which are better adapted than crops to climatic adversities [3]; this activity has a strong influence on the local and regional economy [2].

### Study area

The study was carried out in the communities of Barrocas and Cachoeira, which are located in the rural zone around Soledade city (7°03'26" south latitude and 36°21'46" west longitude), in the state of Paraíba, in Northeastern Brazil. The region has a hot, semi-arid climate (BShs according to Köppen), with an average annual rainfall of 300 mm^3^[25].

The Cachoeira and Barrocas communities are 14 and 18 km from the center of Soledade, respectively, and approximately 4.5 km from each other [22, 26]. These communities have been selected based on prior ethnobotanical studies that have shown the population using vegetation resources for several purposes, including animal feeding [22, 26].

In Barrocas, 12 farms are registered and are located apart from each other with mostly rural properties. The properties range from 70 to 450 ha in area and are primarily used for animal breeding and the cultivation of forage cactus (*Opuntia ficus-indica* (L.) Mill.), corn (*Zea mays* L.) and beans (*Phaseolus vulgaris* L.). In Cachoeira, a total of 22 residences are registered close to each other, thereby forming a small village. Most of the residences have a backyard with space for animal breeding. The agricultural activities are shared in a land space of ordinary use, with approximately 70 ha devoted to subsistence agriculture and livestock [22, 26].

### Ethnobotanical survey

The ethnobotanical survey was authorized by the Ethics Committee for Research Involving Human Beings of the Federal University of Pernambuco (register SISNEP FR – 260099 and CEP/CCS/UFPE N° 176/09) and was conducted between 2009 and 2011. All informants were guided to sign the free and clear consent form, which was a document that expressly showed that the signer was participating voluntarily.

Data collection used the technique of the free list, i.e., the free citation of species by the informant [23]. In this case, the following question was used: “Do you know plants for animal feeding (cattle, goats and sheep)?” From this list, the family heads responded to more detailed questions relating to knowledge of forage plants, such as the consumption, collection, quantity, nutrition, etc. This step was performed through interviews using semi-structured questionnaires to seek as much information as possible related to each species mentioned, as well as socioeconomic data such as education level, age, profession, monthly income, family composition, residence time and marital status. Finally, a screening was performed among respondents for the selection of key informants, i.e., those who have more knowledge on the subject. A third step of data collection was performed with these informants, in which all questions about each species were repeated to list species by the ordering technique [23]. In addition, questions were asked about plant parts, phenology, time of collection, amount collected, animal preference and nutritional attributes. To ensure the reliability of the information, interviews were conducted in two periods, in the early dry season (September-December 2009) and in the early rainy season (May-June 2010). At that time, participant observation was started, in which practices [23] during the collection activities of plants for animals were recorded, looking at the management of the animal in the pasture as well as diet and plants consumed *in loco*. These observations were captured in field diaries [23].

### Collection of plant material

The collection and local identification of plants was performed with the help of local informers giving guided tours, in which more than one informant was invited to walk through the pastures and forests [23]. The species identification of the material was performed by specialists from the herbarium at the Agronomy Institute of Pernambuco (IPA).

### Data analysis

The data achieved in the interviews were stored in a database in Microsoft Excel (Office 2007), and statistical analyses were performed with the BioEstat software (5.0) [27]. Each identified species was verified with regard to biogeographical origin, with species being considered either as native to South America or as exotic. The nomenclature and authorship of the species were updated in the database from the Missouri Botanical Garden (http://www.tropics.org) and the biogeographical origin through the consultation of the catalogue of plants and fungi, volumes I and II, of the Botanical Garden of Rio de Janeiro [28].

To evaluate the differences between the number of plants used and the number of plants known by the informants (those that effectively have indication of use, but in practice are not used every day by the informant), the Wilcoxon-Mann–Whitney test (P < 0.05) was adopted. The chi-square test (alpha < 0.05) was used to evaluate the differences between the richness of native and exotic plants in the studied communities.

The difference in richness of useful species during the drought and rain seasons was found through the chi-square test. The same test was adopted to verify if there were differences among the species cited in the communities.

The Jaccard coefficient (CJ) was used to verify the resemblance of species mentioned among the communities, adopting the formula CJ = a/a + b + c, where **a** is the number of plants common to both communities; **b** is the total of species unique in the community a and **c** is the total of plants unique in the communities.

The Kruskal-Wallis test was used to evaluate whether there were differences in knowledge between men and women and if social-economic factors influenced the species citations [29]. The data were organized as follows: age (1: ≤30 years old; 2: 31 ≤ 40 years old; 3: 41 ≤ 50 years old; 4: 51 ≤ 60 years old; 5: ≥61 years old), gender (woman; man), family income (1: ≤ 1 minimum wage; 2: > 1 minimum wage), and education level (illiterate, for people who have never attended school; basic education, for people who attended only the first years of school; middle school).

### Socioeconomic profile of the communities

In this study, 44 people were interviewed; 27 of them were in the Cachoeira community, including 16 women aged 24 to 92 years old, of whom 56% were involved in domestic activities and 44% were salaried employees. The men were aged 26 to 65 years old, with 90% engaged in agricultural activities and animal breeding and the rest of them (10%) working in other activities.

With regard to education level, 75% of the population is illiterate; the rest of them (25%) have attended school sometime, but none of them have finished high school. The maximum income in the community does not exceed two minimum wages for 45% of the interviewees, and the other individuals have an income of one minimum wage.

In Barrocas, 70% of the men (9), aged 31 to 71 years old, are farmers and supplement the family income by breeding animals such as swine, horses, goats, sheep and cattle. Regarding women (8), aged 38 to 73 years old, 33% are also farmers, 30% work in several activities for the government and for private employers, and 37% are engaged in domestic activities.

In this community, 50% of people live with a minimum wage as an income, and the others earn just a little more than that minimum wage except for a registered case whose informant earns an average of five salaries as retirement.

## Results

### Local knowledge about fodder plants

**Figure 1:**
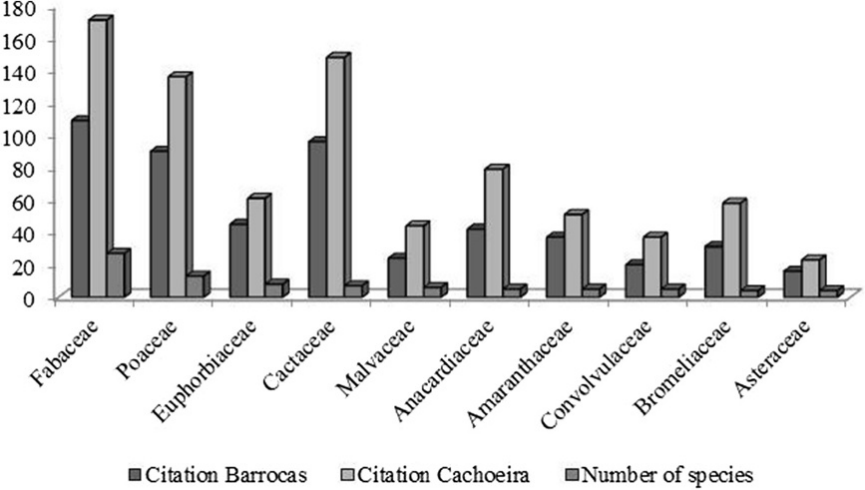
**Families represented by the greatest number of fodder species mentioned in the communities of Cachoeira and Barrocas (Soledade, Paraíba, NE Brazil).**

The respondents displayed vast knowledge, citing more than 1,600 pieces of information about plants (mean 36 ± 16.73 by informant). They identified 136 species that were distributed into 113 genera and 37 families. The species include members of the families Poaceae (13), Fabaceae (13), Euphorbiaceae (8) and Cactaceae (7) (Figure 1), which emphasizes the richness of the species diversity. More than 60% of the information is related to actual use, with a significant representation (978, mean: 8.15 ± 7.3) when compared to other known species with potential in animal nutrition (634, mean = 5.28 ± 5.07), [Z(U) =1.9293; p < 0.05].

The plants indicated by the local experts came from the participant observations. These people accompany the animal throughout the day and observe its preferences; however, in critical periods of drought, the choice is no longer the animal’s but becomes the owner’s, who collect and prepare food for the animal. The best example of this is the use of *Opuntia ficus-indica* (L.) Mill., other cacti such as *Pilosocereus gounellei* (F.A.C. Weber) Byles & G.D. Rowley, bromeliads such as *Bromelia laciniosa* Mart. ex. Schult. f., and the leaves and branches of fruit trees such as *Prosopis juliflora* (Sw.) DC. These are good choices, given their availability, productivity (produced dry weight ratio) and nutritional value.

There was a high similarity (0.75) in the knowledge of residents of the two communities, as there were 99 plant species mentioned in both. Despite the high similarity and geographic proximity between the communities, some plants were registered in only one community: 19 in Barrocas and 18 in Cachoeira. These species had a low frequency of citation (5%), and some of them are cultivated in agroforest backyards, such as *Ricinus communis* L., *Mangifera indica* L., *Moringa oleifera* Lam. and *Anacardium occidentale* L. As shown in Table 1, others corresponded to species of past use, which were usually plants that are no longer found in the region, such as *Licania rigida* Benth., *Parkinsonia aculeata* L. and *Combretum leprosum* Mart.

**Figure 2:**
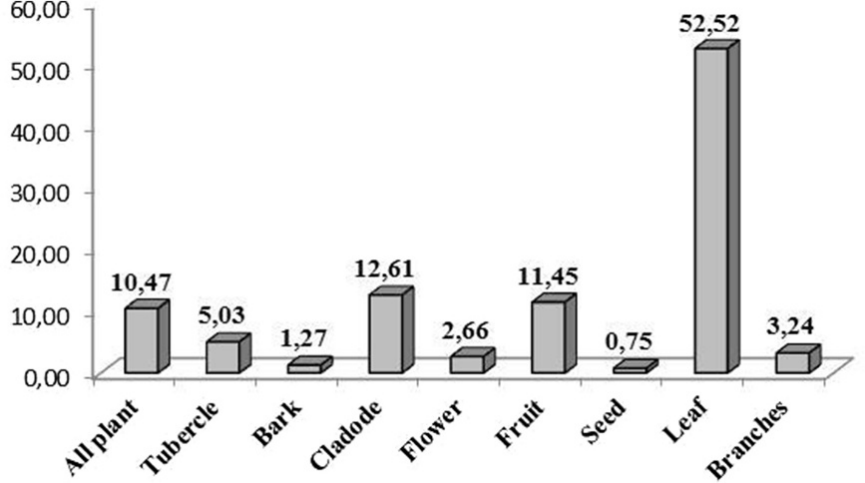
**Percentage of citations of plant parts listed in the diet of ruminants of fodder species mentioned in the Cachoeira and Barrocas communities (Soledade, Paraíba, NE Brazil).**

**Figure 3:**
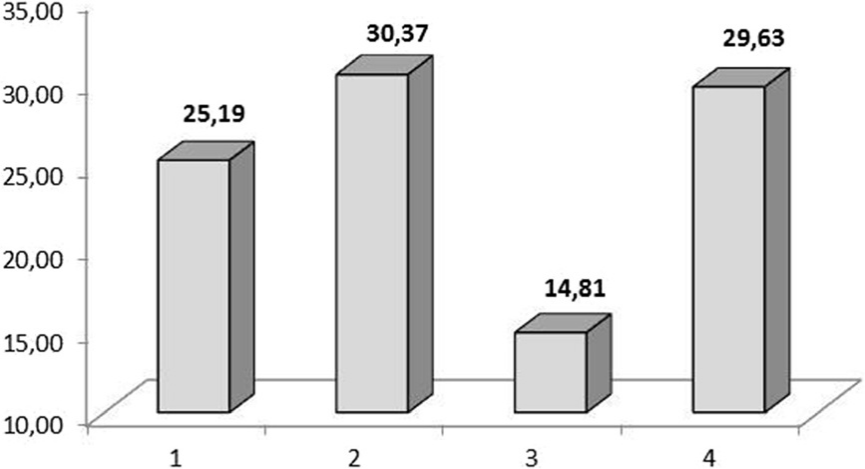
**Percentage of citations of plant parts listed in the diet of ruminants in the Cachoeira and Barrocas communities (Soledade, Paraíba, NE Brazil).** The numerals represent the number of plant parts used.

Finally, for all species, the informants indicated the plant parts preferentially consumed by animals. The parts most frequently consumed were leaves (52.52%), ranging from trees to herbaceous plants, followed by fruits and cladodes (12.61% and 11.45%, respectively) and the entire plant (10.47%) (Figure 2). Considering the number of plant parts, 20% were indicated to have only one part of the plant used for food, while the remaining species had more than two parts mentioned as animal feed (Figure 3).

### Use of native and exotic plants

Seventy identified species were classified as exotic and 67 as native. Thus, with regard to their biogeographical origin, there were no significant differences between native and exotic plants (χ^2^ = 0.06, p > 0.05).

The following native species are highlighted as the most cited: *Pilosocereus gounellei* (F.A.C. Weber) Byles & G.D. Rowley (xique-xique, 85), *Spondias tuberosa* Arruda (umbu, 63), *Tacinga palmadora* (Britton & Rose) N.P. Taylor & Stuppy (Palmatória, 49), *Bromelia laciniosa* Mart. ex. Schult. f. (macambira, 42), *Ziziphus joazeiro* Mart. (juazeiro, 40), *Cereus jamacaru* DC (mandacaru, 39) and *Caesalpinia pyramidalis* Tul. (catingueira, 36). The most cited exotic species were *Amaranthus cruentus* L. (bredo de porco, 48), *Prosopis juliflora* (Sw.) DC. (algaroba, 45), *Cenchrus echinatus* L. (capim, 39) and *Opuntia ficus-indica* (L.) Mill. (cactus, 38) (Table 1).

**Table 1:** **Forage plants mentioned by the interviewed in Barrocas (A) and Cachoeiras (B), in Soledade city - PB,NE Brazil**

Family	Species	Vernacular name	Part consumption	Citations per community		Origin	Life form	Frequency (%)
				A	B			
Agavaceae	*Agave sisalana* Perrine ex Engler	Agave	Lv, Tb	3	2	E	Herb	32.56
Alismataceae	*Echinodorus andrieuxii* (Hook. and Arn.) Small	Golfe	Lv	-	1	N	Herb	2.33
Amaranthaceae	*Alternanthera tenella* Colla	Quebra panela	Lv, P.t.	5	8	N	Herb	34.88
	*Amaranthus spinosus* L.	Bredo de espinho	Lv, P.t.	7	8	E	Herb	30.23
	*Amaranthus cruentus* L.	Bredo de porco	Lv, P.t.	14	19	E	Herb	100
	*Amaranthus viridis* L.	Bredo	Lv, P.t.	5	8	E	Herb	30.23
	*Gomphrena demissa* Mart.	Cama de amancebado	P.t.	1	-	N	Herb	2.33
Anacardiaceae	*Anacardium occidentale* L.	Caju	Lv	1	-	N	Tree	2.33
	*Mangifera indica* L.	Manga	Lv	1	-	E	Tree	2.33
	*Myracrodruon urundeuva* Allemão	Aroeira	Lv, Fr, Se, Br	13	18	N	Tree	72.09
	*Schinopsis brasiliensis* Engl.	Braúna	Lv, Fr, Br	8	17	N	Tree	58.14
	*Spondias tuberosa* Arruda	Umbuzeiro	Lv, Fr, Br	19	44	N	Tree	100
Apocynaceae	*Aspidosperma pyrifolium* Mart.	Pereiro	Lv, Fr, Br	6	17	N	Tree	53.49
	*Acanthospermum hispidum* DC.	Espinho de cigano	Lv, P.t.	3	5	E	Herb	32.56
Asteraceae	*Blainvillea acmella* (L.) Philipson	Bamburrá	Lv, P.t.	6	4	E	Herb	23.26
	*Helianthus annus* L.	Girassol	Lv	3	8	E	Herb	25.58
	*Simsia dombeyana* DC.	Espinho de agulha	Lv, P.t.	4	-	N	Herb	9.3
Bombacaceae	*Ceiba glaziovii* (Kuntze) K. Schum*.*	Barriguda	Lv, Rz	4	10	N	Tree	32.56
	*Pseudobombax marginatum* (A.St.-Hil.) A. Robyns	Imbiratanha	Lv	-	2	N	Tree	4.65
Boraginaceae	*Heliotropium elongatum* (Lehm.) I.M. Johnst.	Crista de peru	P.t.	4	2	N	Herb	13.95
	*Heliotropium indicum* L.	Fedegoso	Lv	11	4	E	Herb	34.88
	*Heliotropium procumbens* Mill.	Mato-azul	P.t.	4	6	E	Herb	23.26
	*Heliotropium tiaridioides* Cham.	Crista de peru	P.t.	4	8	E	Herb	27.91
	*Varronia leucocephala* (Moric.) J.S.Mill.	Maria preta	Lv	1	1	E	Shrub	4.65
Bromeliaceae	*Bromelia laciniosa* Mart. ex. Schult. f.	Macambira rôxa	Lv, Tb	17	25	N	Herb	97.67
	*Encholirium sp.*	Macambira branca	Lv, Tb	5	17	N	Herb	48.84
	*Neoglaziovia variegata* (Arruda) Mez	Caroá	Lv, Tb	8	17	N	Herb	58.14
	*Tillandsia recurvata* (L.) L.	Salambaia	P.t.	1	-	E	Herb	2.33
Cactaceae	*Cereus jamacaru* DC.	Cardeiro/mandacaru	Fr, P.t.	16	23	N	Tree	90.7
	*Melocactus zehntneri* (Britton & Rose) Luetzelb.	Coroa de frade	P.t.	11	15	N	Herb	60.47
	*Opuntia ficus-indica* (L.) Mill.	Palma*	Fr, Cd	14	24	E	Shrub	88.37
	*Pilosocereus gounellei* (F.A.C. Weber) Byles & G.D. Rowley	Xique-xique	Fr, Cd	36	49	N	Shrub	100
	*Pilosocereus pachicladus* F. Ritter*.*	Facheiro	Lv, Cd	8	14	N	Shrub	86.4
	*Tacinga inaenamoema* Britton & Rose	Cumbeba	Fr, Cd.	2	1	N	Shrub	6.98
	*Tacinga palmadora* (Britton& Rose) N. P. Taylor & Stuppy	Palmatória	Fr, Cd	17	32	N	Shrub	100
Capparaceae	*Cynophalla flexuosa* (L.) J. Presl	Feijão-brabo	Lv, Fr, Br	9	15	N	Tree	55.81
Celastraceae	*Maytenus rigida* Mart.	Bom Nome	Lv, Br	2	2	N	Tree	9.3
Chrysobalanaceae	*Licania rigida* Benth.	Oiticica	Lv, Fr	-	1	N	Tree	2.33
Convolvulaceae	*Jacquemontia bahiensis* O'Donell	Amarra cachorro	Lv	3	11	E	Herb	32.56
Combretaceae	*Combretum glaucocarpum* Mart.	Canela de ema	Lv	-	1	N	Tree	2.33
	*Combretum leprosum* Mart.	Mufumbo	Lv, Fr,	2	1	E	Tree	6.98
Commelinaceae	*Commelina erecta* L.	Olho de Santa Luzia	Lv, P.t.	2	-	N	Herb	4.65
	*Commiphora leptophloeos* (Mart.) J.B. Gillett	Amburana/Imburana	Lv,	3	12	E	Tree	34.88
Convolvulaceae	*Evolvulus glomeratus* Nees & C. Mart*.*	Flor azul	Lv, Fr	2	-	E	Herb	4.65
	*Ipomoea batatas* (L.) Lam.	Batata	Lv	-	1	N	Herb	2.33
	*Ipomoe nil* (L.) Roth	Jitirana a	Lv, P.t.	6	8	N	Herb	32.56
	*Ipomoea* sp. 1	Jitirana c	Lv	5	8	-	Herb	30.23
	*Jacquemontia hirsuta* Choisy	Jitirana	Lv, P.t.	8	14		Herb	32.56
	*Merremia aegyptia* (L.) Urb*.*	Jitirana (branca)	Lv, P.t.	17	3	E	Herb	46.51
	*Merremia* sp. 2	Jitirana	P.t.	3	5	E	Herb	18.6
Cucurbitaceae	*Cucumis anguria* L.	Maxixe*	Lv	1	-	E	Herb	2.33
	*Cucurbita* sp.	Jerimum*	Lv	3	2	E	Herb	11.63
	*Cucurbita* sp*.*	Pepino*	Lv	1	1	E	Herb	4.65
	*Momordica charantia* L.	Melão de São Caetano*	Lv, Fr	1	-	E	Herb	2.33
Cyperaceae	*Cyperus uncinulatus* Schrad. ex Nees	Barba de bode	Lv, Fr, P.t.	6	4	E	Herb	23.26
	*Citrullus lanatus* (Thumb.) Matsum. & Nakai	Melancia*	Lv	3	2	E	Herb	11.63
Euphorbiaceae	*Cnidoscolus quercifolius* Pohl	Favela	Lv	-	1	N	Tree	2.33
	*Croton blanchetianus* Baill.	Marmeleiro	Lv, Fr., Br	16	20	N	Tree	83.72
	*Croton heliotropiifolius* Kunth.	Quebra-faca	Lv	1	2	E	Tree	6.98
	*Croton sincorensis* Mart.	Marmeleiro branco	Lv	-	1	N	Tree	2.33
	*Euphorbia tirucalli* L.	Aveloz	Lv	9	8	E	Tree	39.53
	*Ricinus communis* L.	Carrapateira	Lv	1	-	E	Tree	2.33
Fabaceae	*Amburana cearensis* (Allemão) A.C.Sm.	Cumaru	Lv, Fr, Br	-	2	N	Tree	4.65
	*Anadenanthera colubrina* (Vell.) Brenan	Angico	Lv, Fr, Br	3	7	N	Tree	23.26
	*Bauhinia cheilantha* (Bong.) Steud.	Mororó	Lv, Fr, Br	3	5	N	Tree	18.6
	*Calliandra sp.*	Pimenta d'água	P.t.	1	1	-	Herb	4.65
	*Chamaecrista rotundifolia* (Pers.) Greene.	Malícia	Lv	-	2	N	Herb	4.65
	*Capparis hastata* Jacq.	Feijão de boi	Lv	1	-	N	Tree	2,33
	*Chloroleucon mangense* (Jacq.) Britton & Rose	Coronha braba	Fr	-	1	N	Tree	2.33
	*Crotalaria incana* L.	Chocalho de raposa	Lv	1	2	N	Herb	6.98
	*Desmodium glabrum* (Mill.) DC.	Feijão de rolinha	Lv, P.t.	3	5	N	Shrub	23.26
	*Desmodium distortum* (Aubl.) J.F. Macbr*.*	Rapadura de cavalo	Lv, P.t.	5	16	N	Shrub	48.84
	*Erythrina velutina* Willd.	Mulungu	Lv, Fr, Se	3	5	N	Tree	18.6
	*Froelichia humboldtiana* (Roem. & Schult.) Seub.	Ervanço	Lv, P.t.	5	6	N	Herb	25.58
	*Gliricidia sepium* (Jacq.) Kunth ex Walp.	Gliricídia	Lv, Fr, Br	1	1	E	Tree	4.65
	*Glycine max*(L.) Merr.	Soja*	-	-	1	E	Herb	2.33
	*Indigofera suffruticosa* Mill.	Anil	Lv	4	2	E	Herb	13.95
	*Inga sp.*	Ingazeira	Lv, Fr, Se	4	3	-	Tree	16.27
	*Leucaena leucocephala* (Lam.) Dewit	Leucena	Lv	2	5	N	Shrub	16.27
	*Libidibia ferrea* (Mart. ex Tul.) L.P. Queiroz	Jucá	Lv, Fr, Br	2	3	N	Tree	11.63
	*Mimosa ophthalmocentra* Mart. ex. Benth*.*	Jurema de imbira	Lv, Fr, Br	6	8	E	Tree	32.56
	*Mimosa tenuiflora* (Willd.) Poir.	Jurema-preta	Lv	12	12	N	Tree	55.81
	*Parkinsonia aculeata* L.	Turco	P.t.	2	1	N	Tree	6.97
	*Phaseolus vulgaris* L.	Feijão*	P.t.	10	22	N	Herb	74.42
	*Piptadenia stipulacea* (Benth.) Ducke	Jurema branca	P.t.	12	8	N	Tree	46.51
	*Poincianella pyramidalis* Tul.	Catingueira	Lv, Fr, Br	11	23	N	Tree	79.07
	*Prosopis juliflora* (Sw.) DC.	Algaroba	Fr, Br	17	27	E	Tree	100
	*Senna obtusifolia* (L.) H.S. Irwin & Barneby	Fedegoso	Lv, Br	2	2	N	Shrub	9.3
	*Rhynchosia minima* DG.	Feijão de rolinha	Lv	1	3	N	Shrub	9.3
	*Vigna peduncularis var. peduncularis* (Kunth) Fawc. & Rendle	Feijão miúdo	Lv	1	1	N	Herb	4.65
Loasaceae	*Mentzelia aspera* L.	Amor de velho	Br	1	1	N	Herb	4.65
Lythraceae	*Pleurophora anomala* (A.St.-Hill.)Koehne	Vassourinha	Br	1	-	E	Herb	2.33
Malvaceae	*Gossypium hirsutum* L.	Algodão*	Lv, Fr	2	9	E	Shrub	25.58
	*Herissantia crispa* (L.) Brizicky	Mela bode	P.t.	4	5	N	Shrub	20.93
	*Manihot dichotoma* Ule	Maniçoba	Lv, Fr	8	15	N	Tree	53.49
	*Melochia tomentosa* L.	Malva rôxa	Lv, P.t.	4	-	E	Herb	9.3
	*Pavonia cancellata* Cav.	Jitirana de boi	Lv	1	-	N	Herb	2.33
	*Sida acuta* Burm. f.	Malva relógio	Lv, P.t.	4	2	N	Herb	13.95
	*Sida galheirensis* Ulbr.	Malva amarela	P.t.	17	24	N	Shrub	95.35
	*Sida rombifolia* L.	Malva-preta	P.t.	-	1	E	Shrub	2.33
Moringaceae	*Moringa oleifera* Lam.	Muringa	Lv, Fr, P.t.	2	-	E	Tree	4.65
Myrtaceae	*Psidium guajava* L.	Goiaba	P.t.	1	-	N	Tree	2.33
Nyctaginaceae	*Boerhavia difussa* L.	Pega-pinto	P.t.	8	13	E	Herb	27.91
Plantaginaceae	*Stemodia* sp.	Meladinha	Lv	1	-	E	Herb	2.33
Poaceae	*Anthephora hermaphrodita* (L.) Kuntze	Capim comum/capim native	Lv	12	15	E	Herb	62.79
	*Brachiaria decumbens* Stapf	Capim braquiária*	Lv	3	9	E	Herb	27.91
	*Cenchrus brownii* Roem. & Schult*.*	Carrapicho de cavalo	Lv, P.t.	4	2	E	Herb	13.95
	*Cenchrus ciliares* L.	Capim-buffel	Lv	3	3	E	Herb	13.95
	*Cenchrus echinatus* L.	Capim-carrapicho	Lv	15	24	E	Herb	90.7
	*Chloris barbata Sw.*	Capim pé-de-galinha	Lv	5	8	E	Herb	30.23
	*Chloris orthonoton* Doll.	Capim-de raiz	Lv	6	6	E	Herb	27.91
	*Dactyloctenium aegyptium* (L.) Willd*.*	Capim pé-de-galinha	Lv, P.t.	3	12	E	Herb	34.88
	*Enteropogon mollis* (Nees) Clayton	Capim do mato	Lv	12	5	N	Herb	39.53
	*Leersia hexandra* Sw.	Capim marreco	Lv	8	4	N	Herb	27.91
	*Pennisetum purpureum* Schum	Capim-elefante*	Lv	11	13	E	Herb	55.81
	*Tragus berteronianus* Schult.	Carrapicho de ovelha	Lv	6	10	E	Herb	37.21
	*Saccharum sp.*	Cana-de-açúcar*	Lv	2	2	E	Herb	9.3
	*Sorghum bicolor* (L.) Moench	Milho sorgo*	Lv	6	15	E	Herb	48.84
	*Triticum sp.*	Trigo*	Lv	1	-	E	Herb	2.33
	*Zea mays* L.	Milho*	Lv	14	22	E	Herb	83.72
Portulacaceae	*Portulaca elatior* Mart. ex. Rohrb*.*	Bredo de alecrim	Lv	2	-	E	Herb	4.65
	*Portulaca halimoides* L.	Alecrim do mato	Lv, P.t.	1	-	E	Herb	2.33
	*Portulaca oleracea* L.	Berdruega/Bredo gordo	Lv, P.t.	4	3	E	Herb	16.28
Rhamnaceae	*Zizyphus joazeiro* Mart*.*	Juá	Lv, Fr, Br	14	26	N	Tree	93.02
Rubiaceae	*Borreria verticillata* (L.) G. Mey*.*	Vassourinha de botão	Lv	-	1	E	Herb	2.33
	*Diodia teres* Walter	Mata pasto	Lv	0	2	E	Herb	4.65
	*Richardia grandiflora* (Cham. & Schltdl.) Steud.	Melancia de vaca	Lv, Br	11	13	E	Herb	55.81
Sapindaceae	*Cardiospermum oliveirae* Ferrucci	Pratudo	Lv, Fr, P.t	3	-	E	Herb	6.98
	*Serjania glabrata* Kunth.	Mata cachorro	Lv	-	2	N	Herb	4.65
Sapotaceae	*Sideroxylon obtusifolium* (Humb. ex Roem. & Schult.) T.D. Penn*.*	Quixabeira	Lv, Fr, Br	14	22	N	Tree	83.72
Scrophulariaceae	*Scoparia dulcis* L.	Vassourinha	P.t.	4	1	E	Herb	11.63
Solanaceae	*Nicotiana glauca* R. Graham*.*	Oliveira	Lv, Fr	1	1	E	Shrub	4.65
	*Solanum agrarium* Sendtn	Gogoia	Lv, Fr	-	1	E	Herb	2.33
	*Solanum americanum* Mill.	Erva Moura	Lv, Fr	2	4	N	Herb	13.95
Sterculiaceae	*Waltheria rotundifolia* Schrank	Malva branca	Lv, P.t.	13	20	E	Herb	76.74
Verbenaceae	*Stachytarpheta angustifolia* (Mill.) Vahl	Milho de cobra	P.t.	2	1	E	Herb	6.98

Legend. Plant part consumed: Cl = Cladode; Fl = flower; Fr = Fruits; Br = Branch; Lv = leaves; P.t. = Entire plant; Rz = Rhizome; Se = Seed; Status: N = Native; E = Exotic; Freq. = Frequency of citation.

*cultivated.

**Figure 4:**
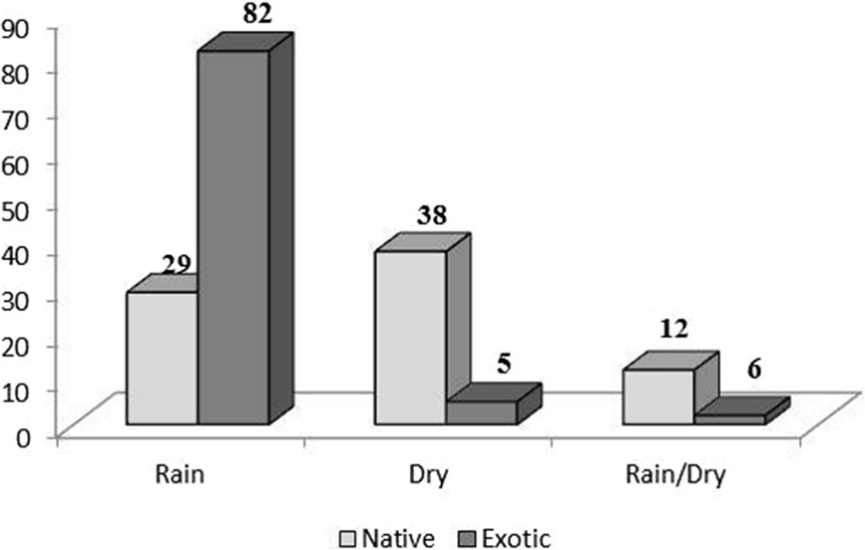
**Number of native and exotic plants used in dry and rainy season, in the communities of Barrocas and Cachoeira (Soledade, Paraíba, NE Brazil).**

It was observed that among the plants native and exotic, there was a strong correlation with the period of the year (χ^2^ = 28.104, *p <* 0.0001); in other words, during the drought season, there were 44 native species and 13 exotic cited, but during the rainy season, there were 25 native plants and 55 exotic mentioned, meaning that the diet of animals can be conditioned by climatic factors (Figure 4). For example, in the rainy season, there were more citations for herbaceous species represented by species of the Poaceae and Fabaceae families. In the dry season, the highlights were the cactus species, such as *P. gounellei*, and some bromeliads, such as *B. laciniosa*, both present year round but limited in the dry season.

**Figure 5:**
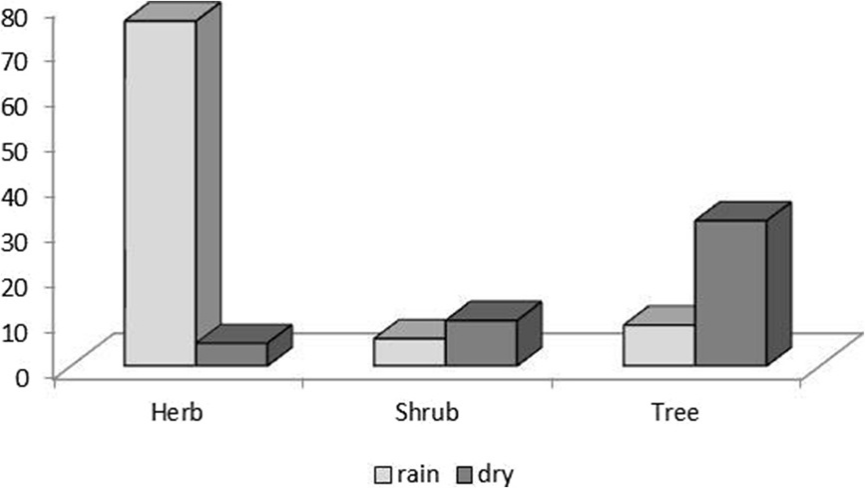
**Diversity of species cited in dry and rainy season according to the plant habit in the communities of Barrocas and Cachoeira (Soledade, Paraiba, NE Brazil).**

The most representative families were Poaceae (43%), Malvaceae *(*10%) and Euphorbiaceae (5%). The most highlighted plants were *Waltheria rotundifolia* Schrank, *Cenchrus echinatus* L., *Dactyloctenium aegyptium* (L.) Willd., *Enteropogon mollis* (Nees) Clayton, *Blainvillea acmella* (L.) Philipson, *Froelichia humboldtiana* (Roem.& Schult.) Seub., *Jacquemontia hirsuta* Choisy and *Merremia aegyptia* (L.) Urb. In addition, the herbaceous plants in the diet of the animals are complemented during the rainy season by the new leaves of woody plants (Figure 5), beyond the fruit, flowers and inflorescence.

### Socioeconomic factors

At the studied communities, the socioeconomic factors, such as age (P > 0.05), income (P > 0.05) and education level (P > 0.05), had no influence on the knowledge of fodder plants, except regarding gender, where men have much more knowledge than women. For men, the average number of known plants was 43 (±10.67) for a total of 926 citations, while for women, there were a total of 647 citations, for an average of 26 (±10.55). The gender difference was statistically significant (H = 11.61, p = 0.0007).

However, it was observed that in Barrocas, the knowledge between men and women (H = 1.33, p > 0.05) did not differ significantly. In other words, in Barrocas, both men and women know and use these resources, while in Cachoeira, the data demonstrated greater knowledge on the part of the men in relation to women (H = 14, p < 0.05).

## Discussion

The results of this research show that there is a wealth of information for feeding ruminants, with respect to the vegetation composition, nutritional value, palatability and the seasonal availability of the species in both communities, which is a finding similar to those of studies in other countries (11, 14, 20). These characteristics, palatability, nutritional value and dry matter productivity, are classified by the community as "sweet plants", "fattening plants" and plants for "fill", respectively, and this rating reflects the perceptions of local experts who deal with animals daily. Although this is not a sophisticated classification, as found by Chettri and Sharma (2009) in India, it signals the abundant knowledge of animal food resources of the participants.

### Local knowledge of forage plants

The informants told the interviewers about a higher diversity of useful plants in the feeding of ruminants, compared to prior lists of fodder plants of the Caatinga [3, 5, 12, 30–33]. Those lists show that the Caatinga has a plant diversity that is relatively similar to the plants used by traditional communities in Africa and Mexico [7, 8, 11, 14].

Among the families most often cited, the grasses and Fabaceae are the most prominent in the literature, and information regarding the potential productivity and nutritional value is abundant, mainly due to the preference of animals for these families (Table 1). The Fabaceae are classified as sweet and fattening plants, (palatable and nutritious) and Poaceae families are classified as palatable and productive, which are highlighted in citations for higher species richness, and are characterized as having high forage potential in Brazilian semiarid areas [30, 32, 34, 35] and in other regions [8, 9, 36, 37].

Locally, the high similarity of local knowledge of fodder resources is a function of the proximity of the communities. On the other hand, the exclusive plants in each community may be reflections of the structural differences of the areas. Groups of species can be common between neighboring areas [7]. For example, Okoli et al. [7] found in three communities of Nigeria that the rearing system could influence the choice of species at each site, i.e., families who kept confined animals demonstrated a greater knowledge about the resources compared with families who raised animals loose in the pasture. However, further investigation is needed to assess the relationship between uses in neighboring areas.

The plant part used in animal feed is an important criterion of the nutritional [38, 39] and ecological [40] point of view. In the case of Caatinga, this richness reflects the local diversity and the high variety of resources involved in the diet [3]. The results may reflect the general nature of ruminants adapted to the region that feed from seedling leaves and tree twigs, a fact which confirms prior research in the region [3, 12, 30]. In other regions, the high abundance of resources for animals has been used to understand and to validate the quality of these resources. Investigations carried out in rural Indian communities have disclosed the preference of the informants for woody resources, especially in places with semi-arid climates [7, 11, 14, 41]. In some cases, following ethnobotanical inquiries, other species have become listed for nutritional characterization, reinforcing the importance of the local knowledge in the characterization of fodder resources [11, 20, 41].

Recent research carried out in traditional communities in underdeveloped countries discloses a scenario similar to that found in this study regarding the high dependence on fodder plants in the maintenance of subsistence livestock [7, 9, 11, 14, 15], as well as emphasizing the importance of local knowledge as a link in the process of the selection of potential plants for a program of sustainable management and conservation of biodiversity [20].

The present study expands the diversity of species not yet shown in the general listings of forage plants from Northeastern Brazil, with the implications considered as positives for a developed approach, contributing to registering plants and to the awareness of potential species that may be investigated in future research under nutrition, biotechnology and ecological.

### Use of native and exotic plants in ruminant feed

In relation to the biogeographical origin of species, most research in semiarid environments reveals a high diversity of exotic plants in the diet of ruminants, whether spontaneous or cultivated in the communities studied. It is common to note *Prosopis juliflora* (Sw.) DC*.*, *Opuntia ficus-indica* (L.) Mill*., Brachiaria decumbens* Stapf and *Chloris orthonoton* Doll. due to their importance in production and nutrition [13, 42, 43].

Native plants are a valuable resource in the communities, as indicated by respondents. These species are distinguished by the availability and nutritional quality to meet the demands of providing animal weight gain and increase in milk production. Further research is needed for these species.

In the rainy season, the herbaceous plants stand out because they are the most abundant resources, represented mainly by exotic species such as *Brachiaria decumbens* Stapf and *Cenchrus ciliaris* L., *Merremia aegyptia* (L.) Urb. [6]. As rainfall decreases, the herbaceous plants are replaced by the woody species of the region.

Both native and exotic species constitute important elements in the studied communities. Some cultivated species, such as *Prosopis juliflora* (sw.) DC., *Opuntia ficus-indica* (L.) Mill., *Brachiaria decumbens* Stapf and *Chloris orthonoton* Doll., have been inserted in the region through governmental incentives; these plants are important regarding their productive and nutritional aspects and, with pastures, increase the diversity of the available resources in the diet of the animals [42].

Native plants constitute a valuable resource, according to the indications of the informants. These species are highlighted because of their availability and nutritional quality for meeting animals’ requirements, providing weight gain and an increase in milk production. In addition to the benefits of these plants, further study should take into consideration the ecological pressures on these species, which deserve greater attention with regard to management issues and sustainability.

### Socioeconomic factors

Local knowledge may be influenced by variables such as gender, income, age and education level. There is a clear differentiation between men and women; this can be associated with the division of existing work in the communities. Women generally manage feeding and family care, and therefore address medicinal, mystic-religious and ornamental plants [44–47]. In the case of the use of fodder, this relation may vary. For example, men dominated the knowledge about fodder plants in the studied communities, suggesting that they are responsible for animal breeding and the collection of plants. A different result is observed in Pakistan [15], where women and children participate in the collection of forage plants and dominate the resources. These variations are important and must be considered in research involving local communities.
